# Supplementary material for: A high-resolution mRNA expression time course of embryonic development in zebrafish
Source: eLife. 2017 Nov 16;6:e30860. doi: 10.7554/eLife.30860 (PMC5690287; doi:10.7554/eLife.30860)
Supplement: Supplementary file 6. [file elife-30860-supp6.zip › biolayout-clusters-files/Cluster221-genes.html]

Cluster221


# Cluster221: Genes

| Ensembl ID | Gene Name | Chr | Start | End | Biotype |
| --- | --- | --- | --- | --- | --- |
| ENSDARG00000099635 | akap8l | 1 | 58441190 | 58530322 | protein\_coding |
| ENSDARG00000030700 | ctps1a | 19 | 16111676 | 16122736 | protein\_coding |
| ENSDARG00000100143 | lrp6 | 4 | 169196 | 195214 | protein\_coding |
| ENSDARG00000024381 | msto1 | 19 | 776399 | 791207 | protein\_coding |
| ENSDARG00000016345 | ncoa5 | 6 | 55417603 | 55425766 | protein\_coding |
| ENSDARG00000024920 | tmem136a | 15 | 22499658 | 22509019 | protein\_coding |
